# Rs884225 polymorphism is associated with primary hypertension by compromising interaction between epithelial growth factor receptor (EGFR) and miR‐214

**DOI:** 10.1111/jcmm.15976

**Published:** 2021-02-26

**Authors:** Fang Luo, Yitian Wu, Qunfang Ding, Yiming Yuan, Weiguo Jia

**Affiliations:** ^1^ The Center of Gerontology and Geriatrics West China Hospital Sichuan University Chengdu China; ^2^ National Clinical Research Center of Geriatrics West China Hospital Sichuan University Chengdu China; ^3^ Department of Laboratory Medicine West China Hospital Sichuan University Chengdu China

**Keywords:** apoptosis, EGFR, microRNA‐214, primary hypertension, rs884225

## Abstract

Genetic variations in the 3′UTR of mRNAs as well as sequences of microRNAs (miRNAs) and long non‐coding RNAs (lncRNAs) can affect gene expression by interfering with the binding between them. In this study, we investigated the role of the following polymorphisms in the risk of hypertension: the 774T > C (rs17337023) polymorphism located in the EGFR 3’ untranslated region (3’UTR), the rs884225 polymorphism located in the sequence of miR‐214, and the single nucleotide polymorphisms (SNPs) rs325797437, rs344501106, rs81286029 and rs318656749 located in the promoter of lncRNA MEG3. Taqman genotyping assays and haplotype analysis tools were used to measure the MEG3 haplotypes and the rs17337023 and rs884225 polymorphisms genotypes. The relationship between MEG3, miR‐214 and EGFR was validated using computational analysis and luciferase assays. Unlike other polymorphisms, only patients grouped according to their rs884225 genotypes exhibited varied EGFR mRNA and protein levels, which indicated that the rs884225 genotype is associated with the expression of EGFR mRNA and protein levels. MiR‐214 was confirmed to bind to MEG3 and 3’UTR of EGFR by showing that the transfection of exogenous miR‐214 significantly down‐regulated the luciferase activity of A549 and H460 cells transfected with wild‐type MEG3 or wild‐type EGFR 3’ UTR. Additionally, MEG3 overexpression inhibited miR‐214 expression while elevating the EGFR mRNA and protein expressions. Meanwhile, MEG3 down‐regulation demonstrated an opposite result, thus establishing the MEG3/miR‐214/EGRF signalling pathway. Our study confirmed that the T > C substitution of rs884225 polymorphism located in miR‐214 binding site in the 3’UTR of EGFR is associated with increased risk of primary hypertension.

## 1 INTRODUCTION

Several cardiovascular conditions, such as vascular remodelling, are induced by high blood pressure. The primary contributors to vascular remodelling are activated vascular smooth muscle cells (VSMC), and different from other muscle cells, VSMC does not undergo terminal differentiation.^1^ Upon vascular injury, VSMC can be converted into an activated or synthetic phenotype from a quiescent or contractile phenotype. In this way, VSMC regains abilities to proliferate and migrate, to secrete matrix proteins, and to lower the expression of smooth muscle contractile proteins including calponin, smooth muscle myosin heavy chain, smooth muscle 22α (SM22α) and α‐smooth muscle actin (α‐SMA).^2^

The proliferation of VSMC is implicated in the occurrence of progressive renal injury in the presence of high blood pressure and the development of vascular stenosis following endothelial damage.^3^, ^4^ Epidermal growth factor (EGF) is a promising cytokine and growth factors involved in the proliferation of VSMC. As a member of the tyrosine kinase family, the EGF receptor (EGFR, also known as HER1) is implicated in the growth and modulation of normal cells.^5^

As a type of short non‐coding RNAs, miRNAs can bind to complementary sequences in the 3’UTR of their target genes to modulate their protein expression.^6^ Diverse cellular processes, including angiogenesis, cell differentiation and cell proliferation, are regulated by miRNAs. Similarly, long non‐coding RNAs (lncRNAs) can also regulate gene expression by affecting the maturation of certain miRNAs and by interfering with the miRNA/mRNA binding. Many studies have shown that in diverse human malignant tumours such as malignant melanoma and ovarian, breast, gastric and pancreatic cancers, the expression of miR‐214 is up‐regulated.^7^, ^8^ Moreover, the expression of EGFR can be elevated by carrying out the T > C substitution in the 774 polymorphism (rs17337023 polymorphism) located in EGFR 3’UTR to increase the risk for bladder cancer. According to the results of bioinformatics analyses, the EGFR 774T > C polymorphism is suspected to be located in an hsa‐miR‐214 binding site in EGFR 3’UTR, thus affecting the risk of carcinogenesis.^9^, ^10^

Many studies suggested that gene expression may be affected by the single nucleotide polymorphisms (SNPs) located in the 3’UTR of target genes of miRNAs, thus affecting the development of diseases in individual patients. It was previously documented that an SNP (rs884225) located in miR‐214 increased the risk for primary hypertension.^11^ Also, renal miR‐214‐3p was reported to play a functional and genetic role in the development of hypertension by targeting eNOS.^12^ And in a previous study,^13^ miR‐214 expression was reported to be highly expressed in PASMCs in patients with hypoxia‐induced pulmonary hypertension, thus promising miR‐214 as a promising diagnostic tool and novel therapeutic target in the management of hypoxia‐induced pulmonary hypertension. Based on the evidence mentioned above, we hypothesized that rs884225 may interfere with the interaction between EGFR mRNA and miR‐214 to affect the risk of primary hypertension. In addition, we also studied the roles of the rs17337023 polymorphism and the haplotypes of lncRNA MEG3 in the development of primary hypertension.

## 2 MATERIALS AND METHODS

### 2.1 Patients

The Human Research Ethics Committees of West China Hospital has approved this research, and all protocols were performed in accordance with the last vision of the Declaration of Helsinki. Written informed consent was obtained from all patients or their first‐degree relatives before the surgery. A total of 890 participants were enrolled in this study, and 5 mL of peripheral blood was collected from each patient. Among those 890 participants, 436 patients with documented hypertension and blood pressure results were recruited into the experimental group, while healthy 454 patients with normal blood pressure were enrolled in the control group.

In addition, we collected lung cancer tissues from 54 lung cancer patients via surgical intervention, and the 54 enrolled lung cancer patients were divided into the following 3 groups according to their genotypes of rs884225 SNP: 1. TT (*N* = 12); 2. TC (*N* = 26); and 3. CC (*N* = 16). Similarly, the 54 enrolled lung cancer patients were divided into the following 3 groups according to their genotypes of rs17337023 of these: 1. TT (*N* = 7); 2. TA (*N* = 32); and 3. AA (*N* = 15). Moreover, the 54 enrolled lung cancer patients were divided into the following 3 groups according to their haplotypes of MEG3: 1. GGGC (*N* = 30); 2. AAAT/GGGC (*N* = 14); and 3. GAAT/GGGC (*N* = 10). Patients diagnosed with secondary hypertension were excluded from this study.

### 2.2 Genotyping

A DNA extraction kit (Huashun, Shanghai, China) was used to isolate the genomic DNA from VSMC according to the instructions of the manufacturer. A TaqMan genotyping kit (Applied Biosystems) was used to perform the genotyping of rs884225, rs17337023, rs325797437, rs344501106, rs81286029 and rs318656749 SNPs in collected clinical samples. Primer Express® Software v.3.0 (Applied Biosystems) was used to determine the genotype of these SNPs. A fluorescein dye with a minor groove binder (MGB) was used to label the 5’ end of probes specific for each allele of the SNPs. A 7900 Fast Real‐Time PCR System (Applied Biosystems) was used to perform the PCR using 96‐well plates. Positive and negative controls were included in each PCR plate to ensure the accuracy of the genotyping. Each test was repeated at least three times.

### 2.3 RNA isolation and real‐time PCR

A MirVana™ miRNA isolation kit (Ambion) was used to extract the total RNA from VSMC according to the instructions of the manufacturer. The extracted RNA was stored in a −80°C freezer in RNase‐free water (Promega UK). A BioTek Power Wave XS (SSi Robotics) spectrophotometer was used to detect the purity, concentration and content of RNA in each sample. A commercial cDNA Synthesis Kit (Invitrogen) was used to synthesize the cDNA, and an EXPRESS SYBR Green qRT‐PCR Kit (Invitrogen) was used to amplify the cDNA in a 20 mL system containing 1.5 mmol/L primer, 10 mL SYBR Green mix, 1.0 mL template cDNA and water. A Mini Opticon Real‐time PCR System (Life Technologies) was used to perform real‐time PCR based on the manufacturer's recommendation. The 2^−ΔΔCT^ method was used to calculate the relative expression of EGFR (Forward primer: 5’‐AACACCCTGGTCTGGAAGTACG‐3’; Reverse primer: 5’‐ TCGTTGGACAGCCTTCAAGACC‐3’) and miR‐214 (Forward primer: 5’‐ TGCCTGTCTACACTTGC‐3’; Reverse primer: 5’‐GAACATGTCTGCGTATCTC‐3’). The expressions of endogenous U6 (Forward primer: 5’‐ CTCGCTTCGGCAGCACA‐3’; Reverse primer: 5’‐ AACGCTTCACGAATTTGCGT‐3’) and GAPDH mRNA (Forward primer: 5’‐ GTCTCCTCTGACTTCAACAGCG‐3’; Reverse primer: 5’‐ ACCACCCTGTTGCTGTAGCCAA‐3’) were, respectively, used as internal control for miR‐214 and EGFR mRNA. Three independent tests were performed.

### 2.4 Cell culture and transfection

DMEM (Dulbecco's Modified Eagle's Medium) (Life Technologies) containing streptomycin (100 mg/mL), penicillin G (100 U/mL) and 10% FCS (foetal calf serum) (Invitrogen) was used to maintain A549 and H460 cells in an atmosphere of 5% CO_2_/95% air at 37°C. When the confluence of cells reached 80%, Lipofectamine 2000 (Invitrogen) was used to transfect 30 nmol/L of miR‐214 mimics (5’‐ ACAGCAGGCACAGACAGGCAGU‐3’), miR‐214 inhibitors (5’‐ACTGCCTGTCTGTGCCTGCTGT‐3’), and EGFR siRNA (3 pairs in total: Pair 1‐sense: 5’‐UGAUCUGUCACCACAUAAUUACGG‐3’ and Pair 1‐antisense: 5’‐CCCGUAAUUAUGUGGUGACAGAUCA‐3’; Pair 2‐sense: 5’‐UUAGAUAAGACUGCUAAGGCAUAGG‐3’ and Pair 2‐antisense: 5’‐CCUAUGCCUUAG CAGUCUUAUCUAA‐3’; Pair 3‐sense: 5’‐UUUAAAUUCACCAAUACCUAUUCCG and Pair 3‐antisense: 5’‐CGGAAUAGGUAUUGGUGAAUUUAAA‐3’), respectively, into the cells according to the manufacturer's instruction. Similarly, pGL3 empty plasmid (as the pGL3 group), pGL3 carrying MEG3 (as the pGL3‐MEG3 group), siRNA negative control (as the NC group) and MEG3 siRNA (as the MEG3 siRNA group) were, respectively, transfected into A549 and H460 cells to study the role of MEG3 expression on the expression of EGFR and miR‐214.

### 2.5 Luciferase assay

The 3'UTR segment containing the wild type of EGFR was amplified and cloned into the pGL3 downstream of the Renilla luciferase gene (pGL3‐EGFR‐3’UTR‐WT), and simultaneously, the 3'UTR segment of EGFR containing the rs884225 minor allele (pGL3‐EGFR‐3’UTR‐MUT) was also amplified and cloned into the same vector downstream of the Renilla luciferase gene. Furthermore, the pGL3‐3'UTR EGFR with either wild‐type and mutant allele was used to transfect the culture cells with or without cotransfection of miR‐214 mimics.

The wild type of MEG3 (pGL3‐MEG3‐WT) or the mutant of MEG3 (the predicted binding sites were replaced with the counter‐complementary sequence: pGL3‐MEG3‐MUT) were amplified before being cloned into the pGL3. Furthermore, either wild‐type or mutant MEG3 was transfected into the A549 and H460 cells with or without cotransfection with miR‐214 mimics.

The expression of firefly luciferase was also normalized to that of internal control Renilla luciferase to ensure the efficiency of transfection. During transfection, Lipofectamine 2000 was used to cotransfect 30 nmol/L of miR‐214 mimics and various pGL3 plasmids of EGFR 3’ UTR into A549 and H460 cells according to the manual of the manufacturer. Similarly, miR‐214 sequences containing different alleles of rs884225 SNP were also amplified through PCR using Taq Polymerase and Taq PCR Mix. Each fragment was then, respectively, cloned into pGL3 vectors downstream of the Renilla luciferase gene. During transfection, Lipofectamine 2000 was used to cotransfect pGL3‐MEG3‐WT, pGL3‐MEG3‐MUT, pGL3‐EGFR‐3’UTR‐WT and pGL3‐EGFR‐3’UTR‐MUT plasmids of miR‐214 into A549 and H460 cells. At 48 after the start of transfection, a dual‐luciferase assay kit (Promega, Madison, WI) was used to measure the luciferase activity of transfected cells following the manufacturer's protocol. The relative levels of protein expression were calculated based on the ratios of Renilla/firefly luciferase activity. Each experiment was performed at least three times.

### 2.6 Western blot analysis

A549 and H460 cells were harvested and washed twice with cold PBS (Invitrogen). A radioimmunoprecipitation assay lysis buffer (Invitrogen) was used to solubilize the cells according to the product protocol, and then, the cell lysates were centrifuged at 12 000 g and 4°C for 10 min to obtain the supernatant. A DC protein assay kit (Bio‐RAD) was used to determine the concentration of proteins in the supernatant. The protein samples were then boiled in the loading buffer to obtain heat‐denatured protein, which was then separated using 5% SDS–PAGE (polyacrylamide gel electrophoresis) and transferred to an Immobilon‐P membrane (Millipore, Bedford, MA) for 2 hours (120 V). PBS containing 0.1% Tween 20 and 5% non‐fat dry milk was used to incubate the membrane for 1 hour to block non‐specific binding. The primary antibody (anti‐EGFR antibody, 1:1000, SCBT, Santa Cruz, CA) was used to incubate the membrane at room temperature for 2 h, and the membrane was then washed twice with PBS containing 0.1% Tween 20. In the next step, a secondary antibody (1:10 000, SCBT, Santa Cruz, CA) was used to treat the membrane for 60 min, and the membrane was then washed twice with PBS containing 0.1% Tween 20. Enhanced chemiluminescence detection reagents (Amersham Biosciences) were used to detect the bound antibody in accordance with the manufacturer's protocol. Each experiment was performed at least three times.

### 2.7 Immunohistochemistry (IHC) assay

The expression of EGFR protein in collected clinical tissue samples of lung cancer was measured using immunohistochemistry assays. In brief, the tissue blocks were sliced into 4 μm sections and blocked with 3% H_2_O_2_. Antigen repair was carried out by heating the sections in a pH 6.0 citrate buffer for 10 min in a pressure cooker. In the next step, the sections were stained at 4°C overnight with anti‐EGFR primary antibodies (1:2000, Abcam), followed by incubation with appropriate HRP‐labelled secondary antibody (1:1000, Abcam) at room temperature for 30 min. After counterstaining with haematoxylin, the positive expression of EGFR in tissue sections was independently analysed and scored by two experienced pathologists.

### 2.8 Statistical analysis

All data were shown as mean ± SEM. SPSS 13.0 statistical software (SPSS Inc) was used to perform all statistical analyses. Student‐Newman‐Kuels (SNK) test and one‐way analysis of variance (ANOVA) were used to carry out multiple comparisons, with the Bonferroni Procedure being utilized as the post hoc test, while Student's t test was used to carry out paired comparisons. Haplotype analysis was evaluated using Haploview (Broad Institute). Specifically, logistic regression analysis was also carried out to study the association between the polymorphisms or haplotype and the risk of primary hypertension. All differences were considered significant when their *P* value was <.05.

## 3 RESULTS

### 3.1 Characteristics of the participants

The demographic and clinicopathological characteristics of the control patients and hypertension patients were shown in Table 1. The control patients and the hypertension subjected appeared to show no differences in terms of age (*P* > .05), sex (*P* > .05) and content of urea (*P* > .05), creatinine (*P* > .05), glucose (*P* > .05), sodium (*P* > .05), potassium (*P* > .05), total cholesterol (*P* > .05) and triglycerides (*P* > .05), although the levels of systolic blood pressure (*P* < .01) and diastolic blood pressure (*P* < .05) were significantly different due to the presence of hypertension. Nevertheless, the above variables were included in the multivariate logistic regression analysis to evaluate their potential effects on the association between the rs884225 polymorphism and the risk of primary hypertension. Subsequently, we conducted a logistic regression test and found that the presence of the minor allele of the rs884225 polymorphism was significantly associated with an increased risk of primary hypertension (the 95% CI was (1.19‐2.21), and OR was 1.62, *P* = .0021). The comparisons of genotype and allele between the control group and the hypertension group were shown in Table 2. Additionally, the demographic and clinicopathological characteristics of lung cancer patients enrolled in this study were shown in Table 3. Again, the 54 lung cancer patients with different haplotypes including GGGC, AAAT/GGGC and GAAT/GGGC appeared to show no differences in terms of age (*P* > .05), sex (*P* > .05) and content of urea (*P* > .05), creatinine (*P* > .05), glucose (*P* > .05), sodium (*P* > .05), potassium (*P* > .05), total cholesterol (*P* > .05) and triglycerides (*P* > .05).

**TABLE 1:** Characteristics of enrolled participants with or without hypertension

Parameter	Control (*N* = 454)	Hypertension (*N* = 436)	*P* value
Age (years)	44.1 ± 5.4	44.6 ± 6.1	.366
Sex (M/F)	335/119	341/95	.636
Urea (mg/dL)	25.5 ± 4.9	26.1 ± 6.2	.318
Creatinine (mg/dL)	1.1 ± 0.3	1.3 ± 0.2	.505
Sugar (mg/dL)	134.5 ± 17.7	135.6 ± 17.2	.968
Sodium (meq/dL)	137.3 ± 20.4	138.7 ± 16.5	.221
Potassium (meq/dL)	3.7 ± 0.3	3.9 ± 0.6	.435
Systolic blood pressure (mmHg)	103 ± 9	152 ± 8	<.01
Diastolic blood pressure (mmHg)	76 ± 5	98 ± 3	<.01
Total cholesterol (mg/dL)	193.2 ± 23.1	189.4 ± 11.3	.716
Triglycerides (mg/dL)	122.5 ± 12.6	126.2 ± 6.9	.270

**TABLE 2:** Genotype and allele comparison between hypertension and control group

Parameter	Control (*N* = 454) (%)	Hypertension (*N* = 436) (%)	OR (95% CI)	*P* value
MEG3				
Haplotype				
GAAT	18 (4)	13 (3)	1.12 (0.78‐1.23)	.365
AAAT	9 (2)	17 (4)		
GGGC	249 (55)	231 (53)		
AAAT/GGGC	72 (16)	74 (17)		
GAAT/GGGC	90 (20)	96 (22)		
GAAT/AAAT	16 (3)	5 (1)		
rs17337023				
Genotype				
TT	54 (12)	65 (15)	1.21 (0.82‐1.42)	.462
AT	272 (60)	257 (59)		
AA	128 (28)	114 (26)		
TT + AT vs. AA	326 (72)	322 (74)	1.18 (0.76‐1.32)	.583
TT vs. AT + AA	400 (88)	371 (85)	1.15 (0.72‐1.29)	.498
Allele				
T	380 (42)	387 (44)	1.38 (0.86‐1.34)	.521
A	528 (58)	485 (56)		
rs884225				
Genotype				
TT	131(29)	87 (20)	1.62 (1.19‐2.21)	
TC	227 (50)	178 (41)		.0021
CC	96 (21)	171(39)		
TT vs. TC + CC	323 (71)	256 (61)	0.83 (0.61‐1.51)	
TT + TC vs. CC	358 (79)	349 (80)	1.82 (1.36‐2.44)	.271
Allele				
T	489 (53)	352 (40)	0.58 (0.48‐0.79)	<.0001
C	419 (47)	520 (60)		<.0001

**TABLE 3:** Characteristics of enrolled lung cancer patients grouped based on rs884225 polymorphism (SBP: systolic blood pressure, DBP: diastolic blood pressure)

Parameter	TT (*N* = 12)	TC (*N* = 26)	CC (*N* = 16)	*P* value
Age (years)	44.5 ± 6.1	44.7 ± 5.1	45.1 ± 5.9	.561
Sex (M/F)	8/4	16/10	9/7	.817
SBP (mmHg)	122.6 ± 15.78	128.6 ± 13.98	133.3 ± 16.32	.189
DBP (mmHg)	82.5 ± 9.76	86.4 ± 10.06	88.8 ± 11.10	.289
Urea (mg/dL)	26.3 ± 6.1	25.5 ± 6.6	25.9 ± 7.1	.450
Creatinine (mg/dL)	1.1 ± 0.2	1.2 ± 0.3	1.1 ± 0.3	.488
Sugar (mg/dL)	135.1 ± 16.7	134.3 ± 19.1	134.3 ± 19.1	.569
Sodium (mq/dL)	138.7 ± 16.7	140.0 ± 14.0	140.0 ± 14.0	.892
Potassium (mq/dL)	4.0 ± 0.3	3.9 ± 0.9	3.9 ± 0.9	.450
Total cholesterol (mg/dL)	191.9 ± 22.4	193.8 ± 17.1	193.8 ± 17.1	.413
Triglycerides (mg/dL)	127.5 ± 18.3	126.8 ± 9.3	126.8 ± 9.3	.555

### 3.2 Determination of MEG3, miR‐214 and EGFR expression in patients carrying different genotypes of rs17337023, rs884225, rs325797437, rs344501106, rs81286029 and rs318656749 SNP

**FIGURE 1:**
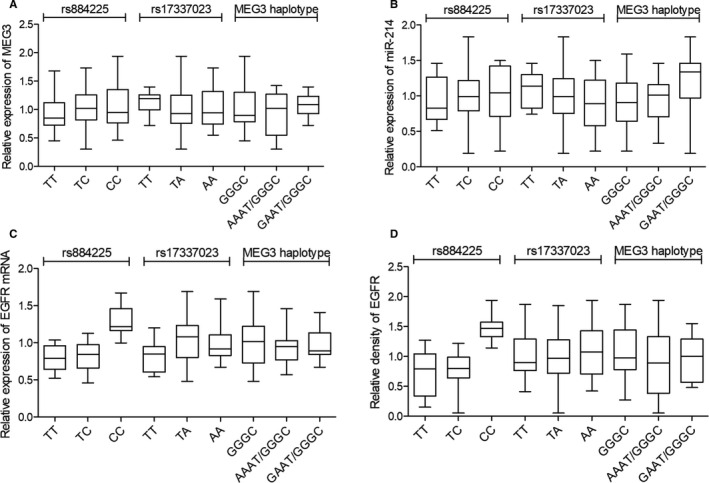
The expression of MEG3, miR‐214, EGFR mRNA and protein in the enrolled lung cancer patients grouped according to different SNP genotypes. A, The expression of MEG3 in lung cancer patients showed was not associated with the genotypes of rs884225 (TT, TC and CC), the genotypes of rs17337023 (TT, TA and AA) nor the haplotypes of MEG3 (GGGC, AAAT/GGGC and GAAT/GGGC). B, The expression of miR‐214 in lung cancer patients was not associated with the genotypes of rs884225 (TT, TC and CC), the genotypes of rs17337023 (TT, TA and AA) nor the haplotypes of MEG3 (GGGC, AAAT/GGGC and GAAT/GGGC). C, The expression of EGFR mRNA in lung cancer patients was not associated with the genotypes of rs17337023 (TT, TA and AA) nor the haplotypes of MEG3 (GGGC, AAAT/GGGC and GAAT/GGGC). However, level of EGFR mRNA was evidently increased in patients carrying CC genotype of rs884225 SNP compared with that in patients carrying TT and TC genotypes of rs884225 SNP. D, Western blot confirmed that the expression of EGFR protein was increased in lung cancer patients carrying CC genotype of rs884225 SNP compared with patients carrying TT and TC genotypes of rs884225 SNP. And EGFR protein expression was not associated with the genotypes of rs17337023 (TT, TA and AA) nor the haplotypes of MEG3 (GGGC, AAAT/GGGC and GAAT/GGGC)

**FIGURE 2:**
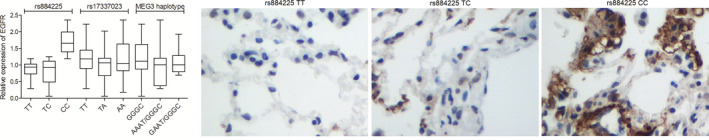
IHC assays confirmed that the expression of EGFR proteins in lung cancer tissues collected from 54 lung cancer patients showed no difference between patients grouped according to the rs17337023 SNP (as TT, TA and AA groups) and haplotypes of MEG3 (as GGGC, AAAT/GGGC and GAAT/GGGC groups). However, patients carrying CC genotype of rs884225 SNP showed evidently increased EGFR mRNA level compared with patients carrying TT and TC genotypes of rs884225 SNP

Real‐time PCR and Western blot were used to further explore the impacts of rs17337023, rs884225, rs325797437, rs344501106, rs81286029 and rs318656749 polymorphisms on the interaction among MEG3, miR‐214 and EGFR 3’UTR in lung cancer tissues collected from 54 lung cancer patients. The 54 enrolled lung cancer patients were divided into different groups according to the genotypes of the rs884225 SNP, the genotypes of the rs17337023 and their haplotypes of MEG3. The results showed no difference of the expression of MEG3 (Figure 1A) and miR‐214 (Figure 1B) among different patient groups. However, in patients grouped according to rs884225 genotypes, the expression of EGFR mRNA (Figure 1C) was 1:1:2.8 and the expression of EGFR protein (Figure 1D) was 1:1:3.2. Meanwhile, in patients grouped according to rs17337023 genotypes or MEG3 haplotypes, no difference in the expression of EGFR mRNA (Figure 1C) and protein (Figure 1D) was found between the groups. Besides, as shown in Figure 2, the IHC results showed no difference in the expression of EGFR protein among patients grouped according to rs17337023 genotypes or MEG3 haplotypes. However, the expression of EGFR proteins in patients grouped according to rs884225 genotypes, the expression of EGFR mRNA was 1:1:3.6.

### 3.3 MiR‐214 binds to MEG3 and 3’UTR of EGFR

**FIGURE 3:**
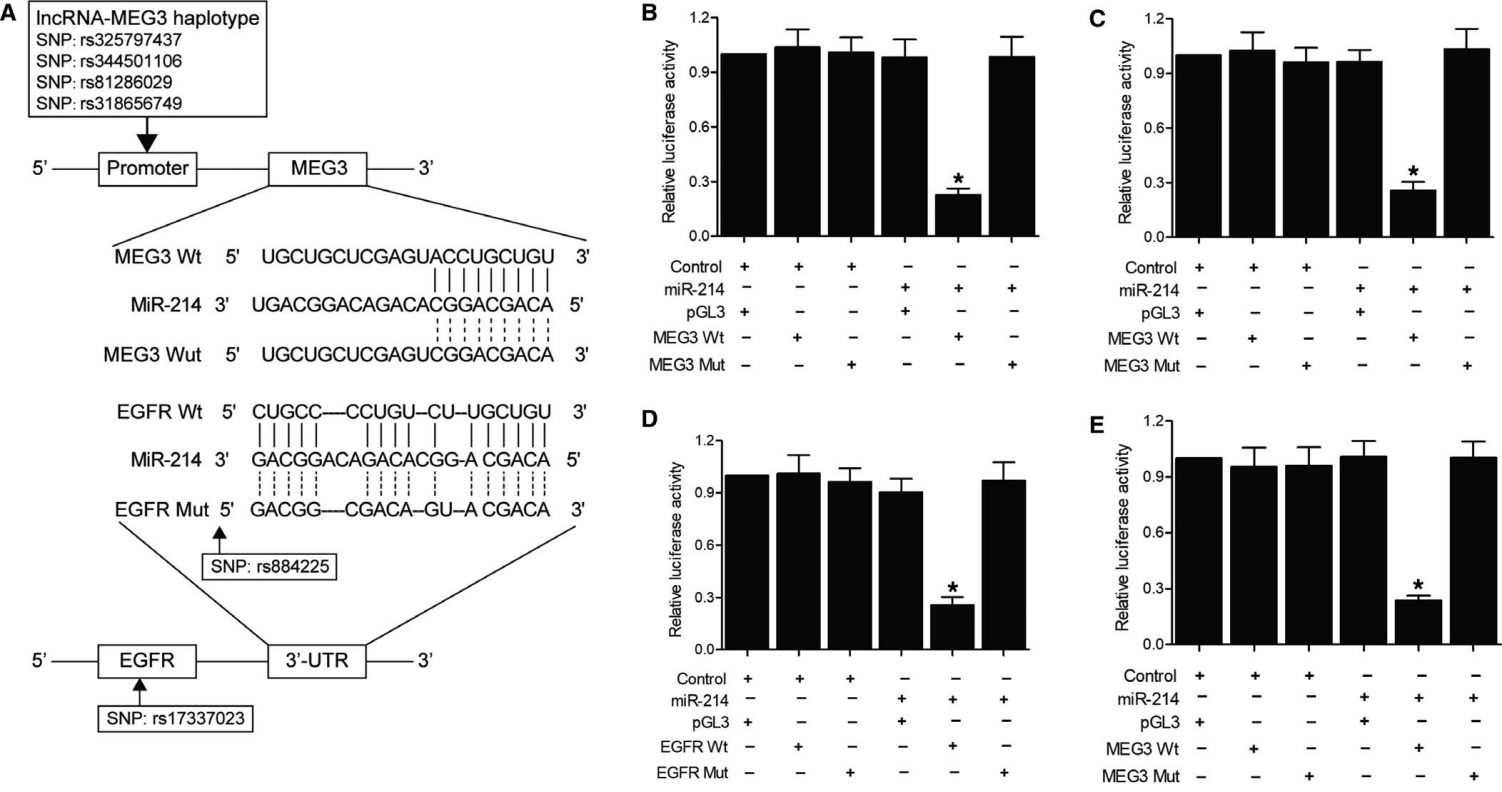
Sequence analysis and luciferase assay analysis upon the interaction between MEG3 and miR‐214, as well as the interaction between EGFR and miR‐214 (*P value < .05, vs. control groups). A, Schematics describing the potential roles of MEG3 haplotype, rs17337023 SNP and rs884225 SNP in the expression of EGFR and miR‐214. B, Only the luciferase activity in A549 cells cotransfected with wild‐type MEG3 and miR‐214 was significantly reduced compared with other cell groups. C, Only the luciferase activity in H460 cells cotransfected with wild‐type MEG3 and miR‐214 was significantly reduced compared with other cell groups. D, Only the luciferase activity in A549 cells cotransfected with wild‐type EGFR 3' UTR and miR‐214 was significantly reduced compared with other cell groups. E, Only the luciferase activity in H460 cells cotransfected with wild‐type EGFR 3' UTR and miR‐214 was significantly reduced compared with other cell groups

Based on the results of computational analysis, the rs17337023 polymorphism is located within a predicted hsa‐miR‐214 binding site in the 3’ UTR of EGFR, while the rs884225 SNP is located in miR‐214 (Figure 3A). In addition, the haplotype of MEG3 containing 4 SNPs, that is rs325797437, rs344501106, rs81286029 and rs318656749 may affect the expression of miR‐214 as well (Figure 3A). To test whether hsa‐miR‐214 targets EGFR 3’UTR and the role of MEG3 in the expression of EGFR and miR‐214, we constructed reporter vectors carrying wild‐type or mutant MEG3 and EGFR 3’UTR, respectively. Subsequently, we transiently cotransfected A549 (Figure 3B) and H460 (Figure 3C) cells with miR‐214 mimics in conjunction with wild‐type or mutant MEG3 (Figure 3B,C), and found that the luciferase activity was only significantly reduced in cells cotransfected with wild‐type MEG3 and miR‐214. Additionally, A549 (Figure 3D) and H460 (Figure 3E) cells were transfected with miR‐214 mimics in conjunction with wild‐type or mutant EGFR 3’UTR, and subsequent luciferase assay only detected evidently reduced luciferase activity in cells cotransfected with wild‐type EGFR 3’UTR in conjunction with miR‐214 compared with the cells in other groups. Therefore, it can be concluded that miR‐214 can bind to both MEG3 and 3’UTR of EGFR.

### 3.4 MEG3 affects the expression of miR‐214 and EGFR

**FIGURE 4:**
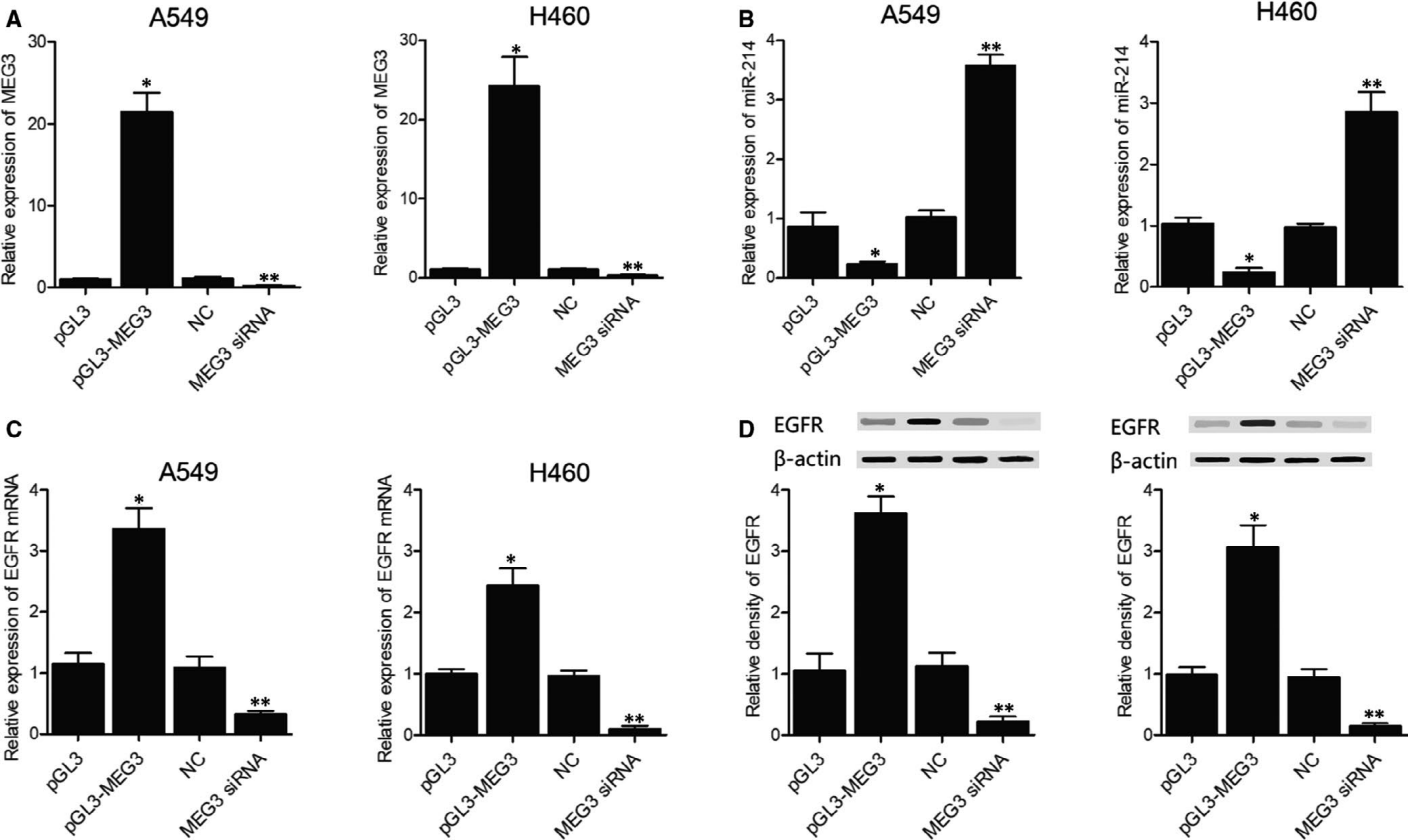
The expression of MEG3, miR‐214, EGFR mRNA and protein in the different A549 and H460 cell groups transfected with pGL3‐MEG3 or MEG3 siRNA in comparison with pGL3 or NC siRNA (*P value < .05, vs. pGL3 group; **P value < .05, vs NC group). A, The expression of MEG3 in different groups was measured by real‐time PCR. B, The expression of miR‐214 in different groups was measured by real‐time PCR. C, The expression of EGFR mRNA in different groups was measured by real‐time PCR. D, The expression of EGFR proteins in different groups was measured by Western blot

To study the effect of MEG3 on the expression of miR‐214 and EGFR, A549 and H460 cells were, respectively, established into four groups: 1. pGL3 in which cells were transfected with empty vector; 2. pGL3‐MEG3 in which cells were transfected with vectors carrying MEG3; 3. NC in which cells were transfected with negative controls; and 4. MEG3 siRNA in which cells were transfected with MEG3 siRNA. The expression of MEG3, miR‐214 and EGFR in different groups was measured by real‐time PCR and Western blot. The results showed that the overexpression of MEG3 by pGL3‐MEG3 (Figure 4A) significantly inhibited the expression of miR‐214 (Figure 4B) but enhanced the expressions of EGFR mRNA (Figure 4C) and protein (Figure 4D). On the contrary, the down‐regulation of MEG3 by MEG3 siRNA (Figure 4A) significantly elevated the expression of miR‐214 (Figure 4B) but reduced the expressions of EGFR mRNA (Figure 4C) and protein (Figure 4D).

## 4 DISCUSSION

Several studies demonstrated that the EGFR signalling pathway plays a significant role in an array of processes of tumorigenesis such as survival, proliferation and apoptosis of tumour cells.^14^, ^15^ In 2000, Mendelsohn and Baselga showed that mitogen‐activated protein kinase (MAPK) and phosphatidylinositol‐3 kinase (PI3K)–AKT pathways are two primary downstream pathways to induce above effects after their activation by EGFR.^16^ In 2007, Yamasaki et al demonstrated that persistently active downstream signalling pathways, particularly PI3k/AKT, can render EGFR‐TKI resistance by bypassing the inhibitory effects of EGFR.^17^ It has been reported in many studies that miR‐214 gene, present in q24.3 of chromosome 1, is abnormally expressed in a variety of tumours including lung, breast, pancreatic and cervical cancers.^18^, ^19^ Increasing evidence demonstrated that miR‐214 serves either a tumour‐inhibitory or oncogenic role in a variety of tumours. Some studies also showed that the proliferation of HeLa cells could be inhibited by miR‐214, which could target PTEN and render ovarian cancer cells resistant to cisplatin.^20^ Inhibition of PTEN results in the activation of the PI3K/AKT pathway, which in turn promotes tumour cell survival during the treatment of cisplatin. Additionally, knockdown of miR‐214 decreased the survival of ovarian cancer cells resistant to cisplatin. In this study, we used computational analysis to show that the EGFR 774T > C polymorphism is located within a predicted hsa‐miR‐214 binding site, and subsequent luciferase assay data confirmed EGFR as a target of hsa‐miR‐214.

Human carcinogenesis may be caused by aberrant miRNA expression and dysregulation of some miRNAs.^20^, ^21^, ^22^ Therefore, specific miRNAs and their targets associated with tumorigenesis can be used as diagnostic and therapeutic tools for patients with malignant tumours. Meanwhile, growing evidence revealed that miRNAs play a crucial regulatory role in the expression of tumour suppressors or oncogenes, leading to tumorigenesis and other diseases.^23^, ^24^, ^25^ Saunders et al suggested that the binding of miRNAs to their target sites in the 3’UTR of human genes may be impacted by SNPs located in the ‘seed sequence’ of binding sites.^26^ Studies have identified an important correlation between the polymorphisms in miRNA binding sites and human disorders.^27^, ^28^, ^29^ In a recent study of Chu et al, EGFR binding with miR‐214 was significantly compromised by the 774 T > C SNP (rs17337023) located in the 3’UTR of EGFR, resulting in an increased risk of bladder cancer in a Chinese population.^30^

Previous studies have assessed the correlation of NPC with two SNPs known as rs7201 and rs17337023 located in the MMP2 and EGFR genes, respectively. Neither rs884225 nor rs7201 showed correlation with the elevated risk of NPC. It was reported that there is a correlation between rs7201 SNP and the risk of small vessel infarcts in stroke, and multivariable logistic regression analyses found rs7201 SNP as an independent risk factor of small vessel infarcts in stroke.^31^ Choi et al showed no correlation between the rs17337023 SNP and the risk of lung cancer.^32^ In this study, we reported that the expression of EGFR mRNA and protein was not affected in patients genotyped as TT or TC for its rs884225 SNP, but patients carrying CC genotype of rs884225 SNP all exhibited elevated expression of EGFR. Therefore, the T to C substitution in the rs884225 SNP of miR‐214 could increase the expression of EGFR by interfering with the interaction between miR‐214 and EGFR. We next performed a logistic regression analysis and found that rs884225 was significantly associated with the risk of hypertension (OR was 1.48, and 95% CI was (1.03‐2.14), and the P value was 0.03 among TT, TC and CC groups).

There is limitation about this study. 1. As the normal human tissue samples were not available for this study, we used malignant tissue resected during surgical intervention to verify the regulatory relationship between MALAT1, miR‐214 and EGFR. 2. The sample size for the association study was still relative small; further study with larger scale was warranted to confirm the result of this study.

## 5 CONCLUSION

The findings of this study confirmed that the minor allele of rs884225 SNP was associated with an elevated risk of primary hypertension and the rs884225 SNP may be used as a predictive biomarker for the diagnosis and management of primary hypertension.

## CONFLICT OF INTEREST

The authors report no relationships that could be construed as a conflict of interest.

## AUTHOR CONTRIBUTION

**Fang Luo:** Data curation (equal); Resources (equal); Validation (equal); Writing‐original draft (equal). **Yitian Wu:** Data curation (equal); Methodology (equal); Software (equal); Visualization (equal). **Qunfang Ding:** Formal analysis (equal); Investigation (equal); Software (equal); Validation (equal). **Yiming Yuan:** Data curation (equal); Investigation (equal); Software (equal); Validation (equal). **Weiguo Jia:** Conceptualization (equal); Data curation (equal); Methodology (equal); Project administration (equal); Resources (equal); Supervision (equal); Writing‐original draft (equal).

## Data Availability

The data that support the findings of this study are available from the corresponding author upon reasonable request.
